# Triple Synchronous Urogenital Malignancies of the Bladder, Kidney, and Prostate: Management in a Single Operation

**DOI:** 10.7759/cureus.47107

**Published:** 2023-10-16

**Authors:** Kyler W Perry, Gabrielle Yankelevich, Leah Ashton, Gregory Diorio

**Affiliations:** 1 Urology, Medical University of South Carolina, Charleston, USA

**Keywords:** surgical management, kidney cancer, bladder cancer, prostate cancer, triple synchronous urogenital malignancies

## Abstract

Synchronous occurrence of three primary malignancies is a rare occurrence, and treatment options are often a difficult undertaking. We present a case of a 57-year-old Hispanic male with synchronous urothelial cell carcinoma of the bladder, renal cell carcinoma, and prostate adenocarcinoma. We elected to manage this patient with a single operation. To our knowledge, this is only the second time a reported operation has been performed of this nature, which includes 21 case reports of triple primary genitourinary tumors, 15 of which are reported as synchronous.

## Introduction

Urothelial cell carcinoma (UCC) of the bladder, renal cell carcinoma (RCC), and prostate adenocarcinoma are all commonly treated urologic malignancies. When these malignancies are primary tumors and found within six months of each other, it is termed synchronous multiple primary malignant tumors (MPMTs) [1]. Individual treatment options for each primary malignancy are well delineated, but treatment for multiple malignancies, especially three malignancies, is more complicated, with limited reports in the literature. 

We report a case of synchronous muscle invasive UCC of the bladder (MIBC), chromophobe RCC, and prostate adenocarcinoma, which were treated surgically in one operation with a laparoscopic radical nephroureterectomy and an open radical cystoprostatectomy. To our knowledge, this is the 15th case of synchronous triple urogenital malignancy reported in the literature and only the second to be treated in one surgery [2,3].

## Case presentation

**Figure 1:**
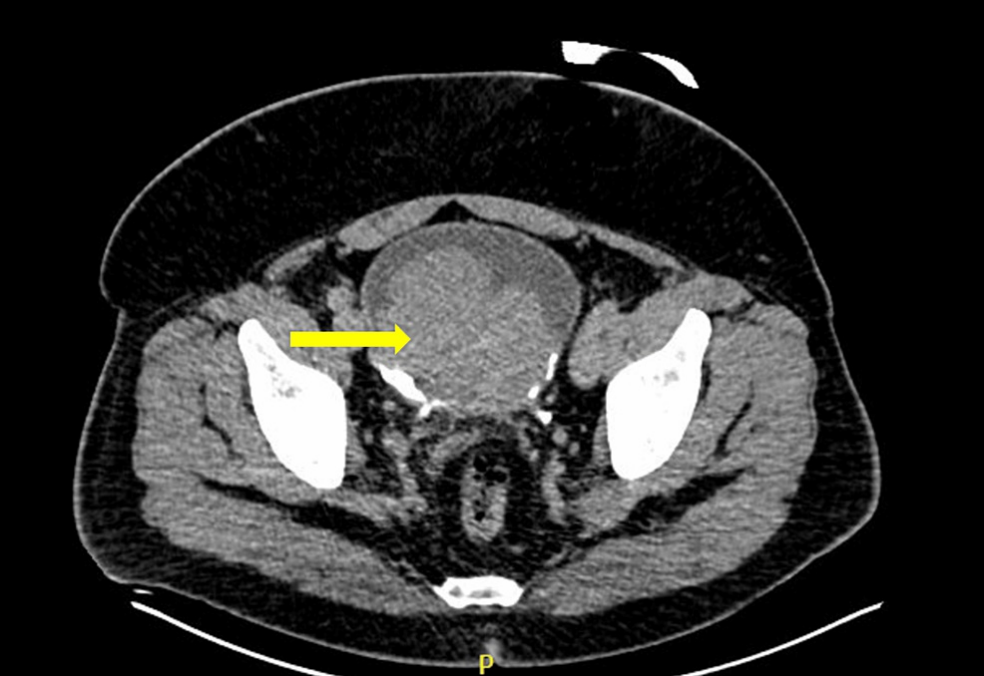
CT urogram with a large tumor burden (yellow arrow) within the bladder

**Figure 2:**
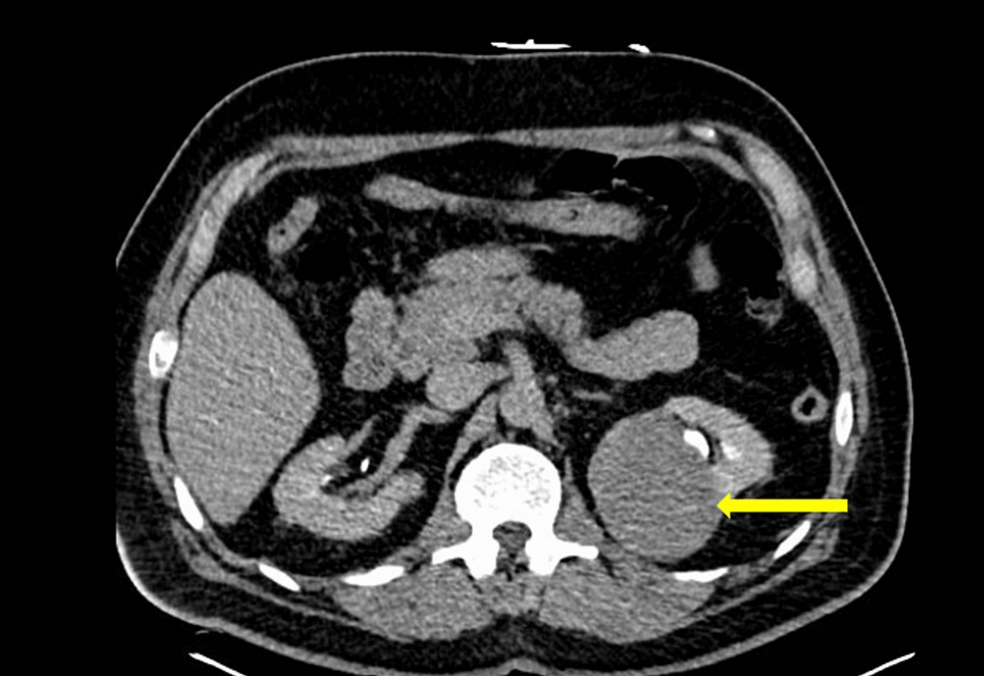
CT urogram with a large left renal mass (yellow arrow) invading the collecting system

**Figure 3:**
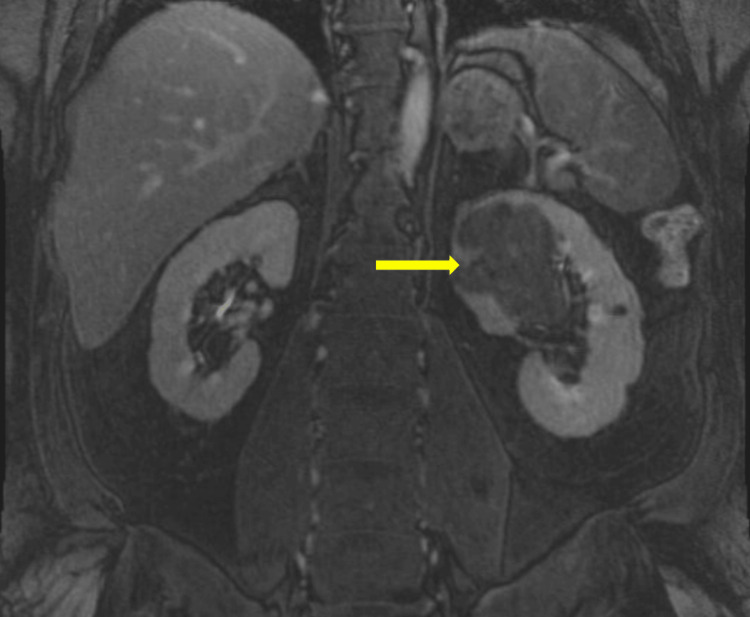
MRI T1 of the left renal mass (yellow arrow) invading the collecting system

A 57-year-old Hispanic male, who could only speak Spanish, with no known past medical history and a two-pack-year smoking history, presented to the emergency room with dyspnea on exertion, fatigue, chest pain on exertion, and gross hematuria for approximately one year. Labs were notable for hemoglobin of 3.8 and creatinine of 1.9 (unknown prior baseline). A computed tomography urogram was obtained, showing an 8.8 x 6.8 cm multi-lobulated enhancing mass nearly filling the bladder and a 7.8 x 6.3 cm heterogeneous mass in the left superior renal calyx (Figures 1, 2). Abdominal magnetic resonance imaging (MRI) was obtained to classify the renal mass, which was significant for a left upper pole mass that was suspicious for urothelial carcinoma due to its nearness to the collecting system (Figure 3). A pelvic MRI was not obtained preoperatively. 

The patient was stabilized with blood transfusions and taken for a transurethral resection of bladder tumor (TURBT) the following day for tissue diagnosis and persistent hematuria requiring continuous bladder irrigation (CBI). Intraoperative findings noted a large tumor burden present after three hours of resection. The patient required CBI after the operating room despite the use of both a bipolar loop and a bipolar button for hemostasis.

The pathology returned as invasive high-grade UCC (at least HgT1), indeterminate for muscle invasion. Given persistent hematuria requiring transfusions and CBI, a large tumor burden not amenable to endoscopic resection, and concern for left upper tract urothelial carcinoma, the decision was made between the patient, urology, and medical oncology to proceed with surgery before possible chemotherapy. 

The patient underwent open radical cystoprostatectomy with ileal conduit, left laparoscopic radical nephrectomy, left adrenalectomy, and bilateral pelvic lymphadenectomy. The nephroureterectomy was performed laparoscopically, tracing the ureter down to the bladder, then converted as planned to an open approach for the cystoprostatectomy, and the specimen was removed en bloc. An adrenalectomy was also performed due to the proximity of the tumor and the high risk of recurrence. An intra-operative frozen section of the urethral margin was benign, so a urethrectomy was not performed. 

The final pathology resulted in primary bladder, prostate, and renal malignancies. Bladder pathology was pT3a pN0 high-grade UCC. Kidney pathology was pT3 pN0 chromophobe RCC. Prostate pathology was incidentally found to be Gleason grade group 2 (3+4) acinar adenocarcinoma, with an estimated 1% of the prostate involved by the tumor. The patient did not have a preoperative PSA.

Oncology recommended somatic next-generation sequencing and germline testing for his bladder cancer, as well as gemcitabine/cisplatin split-dose treatment. The patient was unfortunately lost to follow-up and re-presented after 1.5 years. Surveillance imaging was without disease recurrence, and the patient declined further somatic/germline testing and adjuvant chemotherapy. 

## Discussion

Multiple primary malignant tumors (MPMTs) have long been documented throughout medical literature and are classified under specific criteria [1]. MPMTs may be further classified as synchronous or metachronous. Synchronous MPMTs are defined as primary tumors occurring within six months of one another, while metachronous MPMTs occur within six months of one another [1]. In the present case, all tumors were identified at a single visit, and thus, this represents an example of a synchronous triple primary cancer in the urogenital tract. 

The prevalence of MPMTs varies across multiple studies, ranging from 0.734% to 11.7% [4]. Of these, malignancies occurring in the urinary tract have the highest frequency [5]. It is probable that the increased ability to screen for urinary tract tumors with advancing technology is responsible for this higher percentage. Nonetheless, the prevalence of MPMTs decreases with increasing primary tumors, such that MPMTs with two primary malignancies are more common than those with three [6]. Patients with triple primary tumors make up only 0.5% of MPMT cases [6]. Even further, those that are synchronous have a lower incidence rate, as the criteria require that the tumors occur within six months of each other [7]. While there is no specific data on the incidence of triple urogenital malignancy, namely RCC, UCC, and prostate cancer, there have only been 21 cases previously reported. Of these cases, 15 have met the criteria for being synchronous MPMTs. 

It is well described that patients who present with synchronous MPMTs have a worse prognosis than those with metachronous MPMTs, and thus, treatment must be aggressive [7]. In all but one of the case reports of synchronous urogenital cancers, treatment is handled across multiple surgical steps, as subsequent tumors may arise anywhere from one to six months later [2,3]. Surgical management typically involves a cystoprostatectomy with a radical or partial nephrectomy or nephroureterectomy [8,9]. Only one previous case managed the synchronous triple primary cancers in one operation, performing a simultaneous nephroureterectomy and cystoprostatectomy [2]. To our knowledge, we present the second case to do so. Of note, one previous case utilized hormone therapy for treatment in addition to surgical management [3].

There are several specific risk factors that may predispose patients to malignancy, including radiation, smoking or drinking history, family history, and genetics. This patient did not have a history of drinking, had a minimal smoking history, and did not have a family history of urologic cancers. He had no history of radiation. Therefore, it is not believed that these played a role in his cancer. Genetic determinants certainly play a role, with mutant p53 expression being correlated with worsening outcomes in RCC, bladder cancer, and prostate cancer, while HER-2 expression has been correlated with worsening outcomes in prostate cancer [3]. Further, it has been shown that patients with prostate carcinoma have a higher incidence of bladder cancer and vice versa, suggesting a likely genetic component among MPMTs in the urogenital tract [10].

Lastly, it is important to discuss the social determinants of health and healthcare disparities that led to the challenges in this case. The patient did not have insurance and could only speak Spanish, which likely contributed to the delay in seeking medical care. According to a large retrospective analysis by Hasan et al. for patients with bladder cancer, patients without insurance were found to be independently associated with a diagnosis at a more advanced stage [11]. Uninsured patients were 1.22 times more likely to be diagnosed with muscle-invasive bladder cancer [11]. 

Though a neobladder or continent catheterizable channel would be a reasonable option for this pathology, these were not offered due to financial concerns with obtaining catheters and concern for loss of follow-up. Additionally, though neoadjuvant chemotherapy was discussed with oncology and the patient, the patient had significant hematuria requiring transfusions, and thus disease control was necessary. After discharge, the patient was lost to follow-up for 1.5 years before re-presenting, despite multiple attempts to reach the patient. Moreover, unfortunately, due to poor appointment compliance, the patient did not obtain financial approval for somatic/germline testing, which adds an additional challenge in terms of future treatments if needed. Ultimately, it is important to involve a multidisciplinary team (urologists, oncologists, social workers, home health nursing, etc.), as in this patient's case, to help mitigate barriers to healthcare. 

## Conclusions

UCC of the bladder, RCC, and prostate adenocarcinoma are all commonly treated urologic malignancies. When there are multiple primary urogenital malignancies, treatment is more difficult. We report a case of synchronous triple urogenital malignancies treated surgically in one operation with a laparoscopic radical nephroureterectomy and an open radical cystoprostatectomy. With primary cancers in the urogenital tract, it is important to be aware of the potential for other primary cancers and their presentation. If triple urogenital cancers are found, management options may include surgical treatment of all cancers in a single surgical operation. 

## References

[1] Pan SY, Huang CP, Chen WC (2022). Synchronous/metachronous multiple primary malignancies: review of associated risk factors. Diagnostics (Basel).

[2] Gatto A, Falvo L, Sebastiani S, Roncolini G, Pinna G (2009). Triple synchronous tumours of the urinary system with different histologies: a case report. Chir Ital.

[3] Kurose H, Ueda K, Nakiri M, Matsuo M, Suekane S, Igawa T (2020). Synchronous primary triple urogenital malignant tumors of kidney, prostate and bladder. Urol Case Rep.

[4] Demandante CG, Troyer DA, Miles TP (2003). Multiple primary malignant neoplasms: case report and a comprehensive review of the literature. Am J Clin Oncol.

[5] Liu Z, Liu C, Guo W, Li S, Bai O (2015). Clinical analysis of 152 cases of multiple primary malignant tumors in 15,398 patients with malignant tumors. PLoS One.

[6] Pérès YP, Almeida GL, Busato W (2018). Triple primary malignant tumors of bladder, prostate and lung: case report. J Clin Case Rep Rev.

[7] Friedrich RE (2007). Primary and second primary cancer in 649 patients with malignancies of the maxillofacial region. Anticancer Res.

[8] Pradhan D, Parwani A, Dhir R (2016). Synchronous and metachronous triple primary urogenital cancer: a case report. Am J Clin Pathol.

[9] Tiwari P, Tripathi A, Bansal P, Vijay M, Gupta A, Kundu AK (2012). Synchronous primary cancers of urinary bladder and kidney and prostate. Saudi J Kidney Dis Transpl.

[10] Singh A, Kinoshita Y, Rovito PM Jr (2008). Higher than expected association of clinical prostate and bladder cancers. J Urol.

[11] Hasan S, Lazarev S, Garg M (2023). Racial inequity and other social disparities in the diagnosis and management of bladder cancer. Cancer Med.

